# The Contribution of Cytosolic Group IVA and Calcium-Independent Group VIA Phospholipase A_2_s to Adrenic Acid Mobilization in Murine Macrophages

**DOI:** 10.3390/biom10040542

**Published:** 2020-04-03

**Authors:** Patricia Monge, Alvaro Garrido, Julio M. Rubio, Victoria Magrioti, George Kokotos, María A. Balboa, Jesús Balsinde

**Affiliations:** 1 Instituto de Biología y Genética Molecular, Consejo Superior de Investigaciones Científicas (CSIC), Universidad de Valladolid, 47003 Valladolid, Spain; pmonge@ibgm.uva.es (P.M.); agarrido@ibgm.uva.es (A.G.); jrubio@ibgm.uva.es (J.M.R.); mbalboa@ibgm.uva.es (M.A.B.); 2 Centro de Investigación Biomédica en Red de Diabetes y Enfermedades Metabólicas Asociadas (CIBERDEM), 28029 Madrid, Spain; 3 Department of Chemistry, National and Kapodistrian University of Athens, Panepistimiopolis, 15771 Athens, Greece; vmagriot@chem.uoa.gr (V.M.); gkokotos@chem.uoa.gr (G.K.)

**Keywords:** adrenic acid, arachidonic acid, mass spectrometry, lipid signaling, inflammation, phospholipase A_2_, monocytes/macrophages

## Abstract

Adrenic acid (AA), the 2-carbon elongation product of arachidonic acid, is present at significant levels in membrane phospholipids of mouse peritoneal macrophages. Despite its abundance and structural similarity to arachidonic acid, very little is known about the molecular mechanisms governing adrenic acid mobilization in cells of the innate immune system. This contrasts with the wide availability of data on arachidonic acid mobilization. In this work, we used mass-spectrometry-based lipidomic procedures to define the profiles of macrophage phospholipids that contain adrenic acid and their behavior during receptor activation. We identified the phospholipid sources from which adrenic acid is mobilized, and compared the data with arachidonic acid mobilization. Taking advantage of the use of selective inhibitors, we also showed that cytosolic group IVA phospholipase A_2_ is involved in the release of both adrenic and arachidonic acids. Importantly, calcium independent group VIA phospholipase A_2_ spared arachidonate-containing phospholipids and hydrolyzed only those that contain adrenic acid. These results identify separate mechanisms for regulating the utilization of adrenic and arachidonic acids, and suggest that the two fatty acids may serve non-redundant functions in cells.

## 1. Introduction

Arachidonic acid (*cis*-5,8,11,14-eicosatetraenoic acid, AA), a fatty acid of the *n*-6 series, is the major polyunsaturated fatty acid present in cells of the innate immunity [1]. Cleavage of AA-containing membrane phospholipids by phospholipase A_2_ (PLA_2_) enzymes during activation results in substantial release of free AA. The free fatty acid is then metabolized by cyclooxygenases, lipoxygenases, or cytochrome P-450 enzymes into numerous oxygenated metabolites, collectively called the eicosanoids, which have key roles in inflammation [2,3,4,5,6]. The eicosanoids participate in immune regulation by influencing the activation of innate immune phagocytic cells at various levels, including differentiation and migration, phagocytic capacity, and cytokine production [7,8,9,10].

**Scheme 1:**
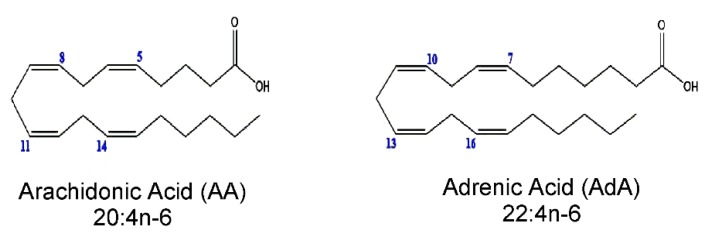
Structure of arachidonic acid (AA) and adrenic acid (AdA). The positions of the carbons at which double bonds occur are numbered. Note that all double bonds are in the *cis* configuration.

Innate immune cells also contain significant quantities of another *n*-6 fatty acid, adrenic acid (*cis*-7,10,13,16-docosatetraenoic acid, AdA), which is the 2-carbon elongation product of AA [1] (Scheme 1). Similar to AA, AdA can also be metabolized via cyclooxygenase, lipoxygenase, and cytochrome P-450 pathways [11,12,13,14], giving rise to a number of oxygenated metabolites. Interestingly, a recent study using a mouse model of steatohepatitis reported elevated levels of free AdA in plasma and liver [15]. These data raised the possibility that alterations in the homeostatic mechanisms regulating AdA levels may be related to pathophysiological states [15]. Regarding the role of AdA in innate immune cells, it has been shown that the fatty acid is able to dampen inflammation by blocking leukotriene B_4_ formation by neutrophils and enhancing phagocytosis by macrophages [16]. These effects are consistent with a pro-resolving mediator function for AdA in osteoarthritis [16].

Recent work from our laboratory has taken advantage of mass-spectrometry-based lipidomic approaches to define at a molecular level the mechanisms regulating AA mobilization from phagocytic cells responding to stimuli of the innate immune response [17,18,19,20,21,22,23,24,25,26,27]. Our work has highlighted not only the importance of multiple PLA_2_ enzymes in the process, but also of the mechanisms regulating the reacylation of the liberated fatty acid back into phospholipids and the remodeling that places the AA in the appropriate cellular lipid pools. These findings have raised the intriguing possibility that not all AA pools may be reachable by the PLA_2_ form involved in its mobilization. For instance, release of lipoxygenase products by ionophore-activated human neutrophils [28] and zymosan-stimulated mouse peritoneal macrophages [22] appears to be associated with AA mobilization from choline-containing phospholipids (PC), not ethanolamine-containing phospholipids (PE) or phosphatidylinositol (PI). Thus, depending on stimulation conditions, the cellular distribution of AA between various phospholipid locations may also limit eicosanoid synthesis [3,26].

In contrast to AA studies, very little is still known about the mechanisms governing cellular AdA availability. In this study we have analyzed the regulatory features of AdA mobilization in activated macrophages. Our work provides an in-depth examination of AdA homeostasis under pathophysiologically relevant conditions, and suggests that both cytosolic group IVA PLA_2_ (cPLA_2_α) and calcium-independent group VIA PLA_2_ (iPLA_2_-VIA) participate in the process. AdA mobilization shares regulatory features with AA mobilization with regard to cPLA_2_α involvement, but seems to be a more complex process, as it involves participation of a second PLA_2_ that is apparently not involved in AA release (i.e., iPLA_2_-VIA). In addition, our study also suggests that supplementation with AdA does not reduce AA utilization by the cells. Thus, the two fatty acids may play non-redundant biological roles that could be exploited to design selective strategies to control the production of AdA-derived products at the level of their precursor fatty acid.

## 2. Materials and Methods

### 2.1. Reagents

Cell culture medium was from Molecular Probes-Invitrogen (Carlsbad, CA, USA). Organic solvents (Optima^®^ LC/MS grade) were from Fisher Scientific (Madrid, Spain). Lipid standards were from Avanti (Alabaster, AL, USA) or Cayman (Ann Arbor, MI, USA). Silicagel G thin-layer chromatography plates were from Macherey-Nagel (Düren, Germany). The cPLA_2_α inhibitor pyrrophenone [29] was synthesized and provided by Dr. Alfonso Pérez (Department of Organic Chemistry, University of Valladolid, Valladolid, Spain). The iPLA_2_-VIA inhibitors FKGK18 and GK436 were synthesized in the Kokotos laboratory [30,31]. All other reagents were from Sigma-Aldrich (Madrid, Spain).

### 2.2. Cell Culture

Mouse peritoneal macrophages from Swiss mice (University of Valladolid Animal House, 10–12 weeks old) were obtained by peritoneal lavage using 5 mL cold phosphate-buffered saline and cultured in RPMI 1640 medium with 10% (*v*/*v*) fetal bovine serum, 100 U/mL penicillin, and 100 µg/mL streptomycin, as described elsewhere [32,33]. All procedures involving animals were undertaken under the supervision of the Institutional Committee of Animal Care and Usage of the University of Valladolid (No. 7406000), in accordance with the guidelines established by the Spanish Ministry of Agriculture, Food, and Environment and the European Union.

Cells were placed in serum-free medium for 1 h before addition of stimuli or inhibitors. Afterward, they were challenged by the stimuli for the time indicated. Zymosan was prepared exactly as described [32,33]. Only zymosan batches that demonstrated no measurable endogenous PLA_2_ activity, as measured by in vitro assay under different conditions [34,35,36,37], were used in this study. Cell protein content was quantified according to Bradford [38] using a commercial kit (BioRad Protein Assay, Bio-Rad, Hercules, CA, USA).

### 2.3. Gas Chromatography/Mass Spectrometry (GC/MS) Analyses

Total lipids from approximately 10^7^ cells were extracted according to Bligh and Dyer [39], and the following internal standards were added: 10 nmol of 1,2-diheptadecanoyl-*sn*-glycero-3-phosphocholine, 10 nmol of 1,2,3-triheptadecanoylglycerol, and 30 nmol of cholesteryl tridecanoate. Phospholipids were separated from neutral lipids by thin-layer chromatography, using *n*-hexane/diethyl ether/acetic acid (70:30:1, *v*/*v*/*v*) as the mobile phase [40]. Phospholipid classes were separated twice with chloroform/methanol/28% (*w*/*w*) ammonium hydroxide (60:37.5:4, *v*/*v*/*v*) as the mobile phase, using plates impregnated with boric acid [41]. The bands corresponding to the different lipid classes were scraped off from the plate, and fatty acid methyl esters were obtained from the various lipid fractions by transmethylation with 0.5 M KOH in methanol for 60 min at 37 °C [42,43,44,45]. Analyses were carried out using an Agilent 7890A gas chromatograph coupled to an Agilent 5975C mass-selective detector operated in an electron impact mode (EI, 70 eV). The apparatus was also equipped with an Agilent 7693 autosampler and an Agilent DB23 column (60 m length × 0.25 mm internal diameter × 0.15 µM film thickness) (Agilent Technologies, Santa Clara, CA, USA). Data analysis was carried out with the Agilent G1701EA MSD Productivity Chemstation software, revision E.02.00 [42,43,44,45].

### 2.4. Liquid Chromatography/Mass Spectrometry (LC/MS) Analyses of Phospholipids

Lipids were extracted according to Bligh and Dyer [39], and the following internal standards were added: 20 pmol each of 1,2-dimyristoyl-*sn*-glycero-3-phosphoglycerol, 1,2-dilauroyl-*sn*-glycero-3-phosphoethanolamine, 1,2-dimyristoyl-*sn*-glycero-3-phosphoethanolamine, 1,2-di- heptadecanoyl-*sn*-glycero-3-phosphoethanolamine, 1,2-dimyristoyl-*sn*-glycero-3-phosphoserine, 1,2-dimyristoyl-*sn*-glycero-3-phosphate, 1,2-dipentadecanoyl-*sn*-glycero-3-phosphocholine, 1,2-di- heptadecanoyl-*sn*-glycero-3-phosphocholine, and 1,2-dinonadecanoyl-*sn*-glycero-phosphocholine.

The samples were re-dissolved in hexanes/2-propanol/water (42:56:2, *v*/*v*/*v*) and injected into a Thermo Scientific Dionex Ultimate 3000 high-performance liquid chromatograph equipped with an Ultimate HPG-3400SD standard binary pump and an Ultimate ACC-3000 autosampler column compartment (Waltham, MA, USA). Separation was carried using a FORTIS HILIC column (150 × 3 mm, 3 µm particle size) (Fortis Technologies, Neston, UK). The mobile phase consisted of a gradient of solvent A (hexanes/isopropanol 30:40, *v*/*v*) and solvent B (hexanes/isopropanol/20 mM ammonium acetate in water, 30:40:7, *v*/*v*/*v*). The gradient started at 75% A from 0 to 5 min, then decreased from 75% A to 40% A at 15 min, from 40% A to 5% A at 20 min, was held at 5% until 40 min, and then increased to 75% at 41 min. Then, the column was re-equilibrated holding 75% A for an additional 14-min period before the next sample injection [46]. The flow rate through the column was fixed at 0.4 mL/min. The liquid chromatography system was coupled online to an Sciex QTRAP 4500 Mass Spectrometer equipped with a Turbo V ion source and a TurbolonSpray probe for electrospray ionization (AB Sciex, Framingham, MA, USA). Source parameters were set as follows: ion spray voltage, -4500 V; curtain gas, 30 psi; nebulizer gas, 50 psi; desolvation gas, 60 psi; temperature, 425 °C. Phospholipid species were analyzed in scheduled multiple reaction monitoring mode with negative ionization, detecting in Q3 the *m*/*z* of either 303.2 or 331.2, corresponding to AA and AdA, respectively, as [M-H]^−^. Compound parameters were fixed as follows: declustering potential; −45 V (choline glycerophospholipids), −60 V (ethanolamine glycerophospholipids) −30 V (phosphatidylinositol), −50 V (phosphatidylserine), −60 V (phosphatidic acid), −50 V (phosphatidylglycerol); collision energy: −50 V (choline glycerophospholipids), −40 V (ethanolamine glycerophospholipids), −60 V (phosphatidylinositol), −50 V (phosphatidylserine), −45 V (phosphatidic acid), −45 V (phosphatidylglycerol); entrance potential, −10 V; and collision cell exit potential, −8 V. All glycerophospholipids were detected as [M-H]^−^, ions except choline glycerophospholipids, which were detected as [M + CH_3_COO]^−^ ions. Quantification was carried out by integrating the chromatographic peaks of each species and comparing these with the peak area of the internal standard that corresponded to each class.

## 3. Results

### 3.1. Adrenic Acid and Arachidonic Acid Contents of Murine Peritoneal Macrophages

**Figure 1:**
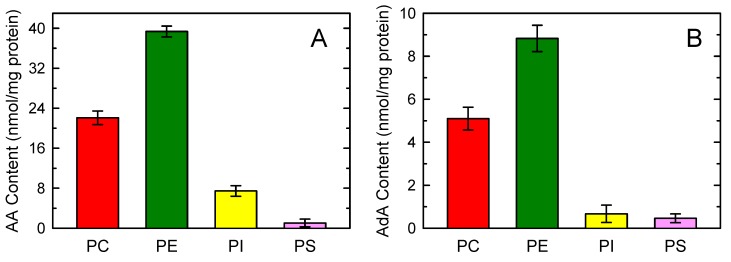
Distribution of AA and AdA between phospholipid classes. The various phospholipid classes were separated by thin-layer chromatography. The distribution of AA (**A**) and AdA (**B**) between choline glycerophospholipids (PC), ethanolamine glycerophospholipids (PE), phosphatidylinositol (PI), and phosphatidylserine (PS) was determined by gas chromatography/mass spectrometry (GC/MS) after converting the phospholipid-bound fatty acids into methyl esters. Results are shown as means ± standard error of the mean (*n* = 3).

Lipid extracts from mouse peritoneal macrophages were analyzed for fatty acid content by GC/MS. Total AA content was 69.9 ± 4.2 nmol/mg cell protein (mean values ± standard error of the mean, *n* = 5), while AdA content was 15.1 ± 1.2 nmol/mg cell protein (mean values ± standard error of the mean, *n* = 5). Both AA and AdA were found almost exclusively in phospholipids. The distribution of AA and AdA between phospholipid classes is shown in Figure 1. Despite the difference in mass between AA and AdA, their distribution between phospholipid classes was remarkably similar, with the majority of both fatty acids being found in ethanolamine glycerophospholipids (PE), followed by choline glycerophospholipids (PC). Minor amounts of both fatty acids were found in phosphatidylinositol (PI) and phosphatidylserine (PS).

**Figure 2:**
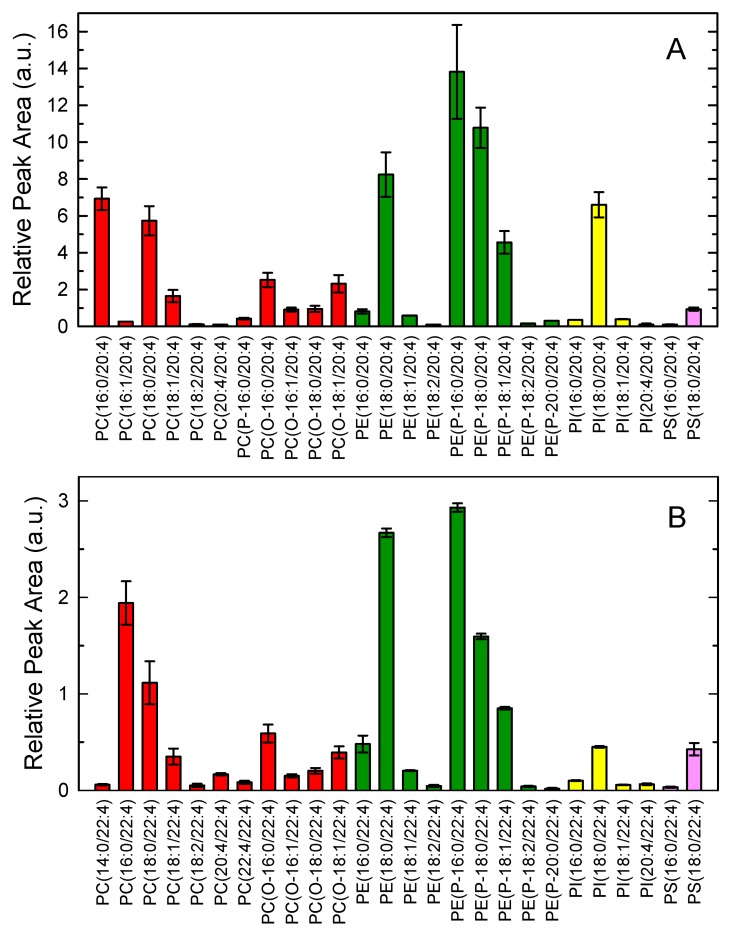
AA and AdA-containing phospholipid molecular species in peritoneal macrophages The profiles of AA- (**A**) or AdA- (**B**) containing PC (red), PE (green), PI (yellow), and PS (pink) species in peritoneal macrophages were determined by liquid chromatography/mass spectrometry (LC/MS). Fatty chains within the different phospholipid species are designated by their numbers of carbons and double bonds. A designation of O- before the first fatty chain indicates that the *sn*-1 position is ether linked, whereas a P- designation indicates a plasmalogen form (*sn*-1 vinyl ether linkage) [47]. Phospholipids containing two ester bonds have no designation. Results are shown as mean values ± standard error of the mean (*n* = 3).

Figure 2 shows the distribution of AA- and AdA-containing phospholipid molecular species, as analyzed by liquid chromatography coupled to tandem mass spectrometry (LC/MS). In agreement with previous estimates [21,22], multiple AA-containing species were detected, with the alkenylacyl and diacyl ethanolamine phospholipid species PE(P-16:0/20:4), PE(P-18:0/20:4), and PE(18:0/20:4) predominating, followed by the diacyl choline phospholipid species PC(16:0/20:4) and PC(18:0/20:4), and the unique inositol phospholipid species PI(18:0/20:4)

Regarding AdA-containing species, the alkenyl acyl and diacyl ethanolamine phospholipid species PE(P-16:0/22:4), PE(P-18:0/22:4), and PE(18:0/22:4)], and the diacyl choline phospholipid species PC(16:0/22:4) and PC(18:0/22:4) also constituted the major cellular AdA reservoirs. Strikingly, the inositol phospholipid species PI(18:0/22:4) was not as prevalent as its AA equivalent, PI(18:0/20:4), was among AA-containing phospholipids. This may suggest that the acyl-CoA acyltransferase using lysoPI as the acceptor [48] shows selectivity for AA over AdA as a substrate.

**Figure 3:**
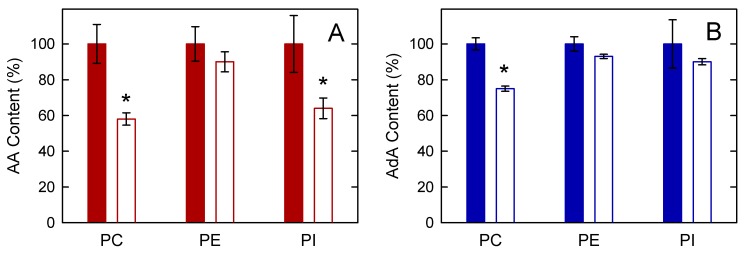
AA and AdA mobilization in zymosan-stimulated macrophages. The cells were unstimulated (colored bars) or stimulated (open bars) with 1 mg/mL zymosan for 1 h. Afterward, total content of AA (**A**) or AdA (**B**) in various phospholipid classes (see x-axis) was measured by GC/MS. To allow for direct comparison between AA and AdA, the fatty acid contents in phospholipid classes in unstimulated cells are given as 100%. Actual mass values can be obtained from Figure 1. Results are shown as mean values ± standard error of the mean. (*n* = 3). Note: * *p* < 0.05, significantly different from the corresponding phospholipid class.

Macrophage stimulation with yeast-derived zymosan markedly decreased the cellular AA content in PC and PI. Despite PE being the major AA-containing class, AA losses from PE did not reach statistical significance (Figure 3A). It should be noted in this regard that during receptor stimulation, AA is known to be transferred from AA-containing PC (1-acyl species) to PE (plasmalogen species) by CoA-independent transacylase; hence, the decline in the amount of AA-containing PE during cellular stimulation may be greatly reduced [22,24,49]. Regarding AdA, decreases in its cellular content were also observed after zymosan stimulation. However the pattern clearly differed in that PC was the only phospholipid class that contributed to AdA mobilization; AdA reductions from PE and PI did not reach statistical significance (Figure 3B).

### 3.2. Defining the Role of Various PLA_2_ Forms in Stimulus-Induced AA and AdA Mobilization

The differential involvement of phospholipid classes in AA and AdA mobilization suggests the existence of separate mechanisms for regulating the availability of each fatty acid. To characterize such mechanisms, we took advantage of the use of selective inhibitors of the two major intracellular PLA_2_ enzymes potentially effecting the fatty acid release in activated peritoneal macrophages, namely group IVA Ca^2+^-dependent cytosolic PLA_2_ (cPLA_2_α) and group VIA Ca^2+^-independent PLA_2_ (iPLA_2_-VIA) [22,50]. The use of selective chemical inhibitors to address the role of intracellular PLA_2_s during cell activation has some advantages over other widely used methods, such as small interfering RNA or cells from knockout mice. With chemical inhibitors, inhibition develops rapidly, which reduces the impact of unspecific effects that could occur over time. Also, no compensatory mechanisms take place that might obscure the interpretation of results [22]. In addition, chemical inhibitors directly target PLA_2_ effects that depend on enzymatic activity, without affecting noncatalytic functions of the enzyme [51]. The inhibitors used in this work are the most potent and selective inhibitors currently available to block cPLA_2_α and iPLA_2_-VIA in cells. Pyrrophenone potently and selectively inhibits cPLA_2_α activity using a number of in vitro assays without detectable effects on other PLA_2_ activities, blocks AA release in mammalian cells in the 0.01–1 μM range [29,52,53], and has been shown to be effective in experimental models of disease involving cPLA_2_α [54,55]. Fluoroketone FKGK18, a selective iPLA_2_-VIA inhibitor, is at least 200-fold more potent for inhibiting iPLA_2_-VIA than cPLA_2_α [30], and is useful for characterizing iPLA_2_-VIA-mediated functions in vivo [31,56,57]. β-Lactone GK436, another iPLA_2_-VIA inhibitor, has been found to be at least 1000-fold more potent for iPLA_2_-VIA than for cPLA_2_α [31]. For comparative purposes with data from the bibliography [22,58,59,60,61,62,63,64], we also used bromoenol lactone (BEL) to inhibit iPLA_2_-VIA. BEL inhibits calcium-independent PLA_2_s and exerts little or no effect on Ca^2+^-dependent enzymes [65,66], albeit it may exhibit off-target effects depending on cell type [67,68,69].

**Figure 4:**
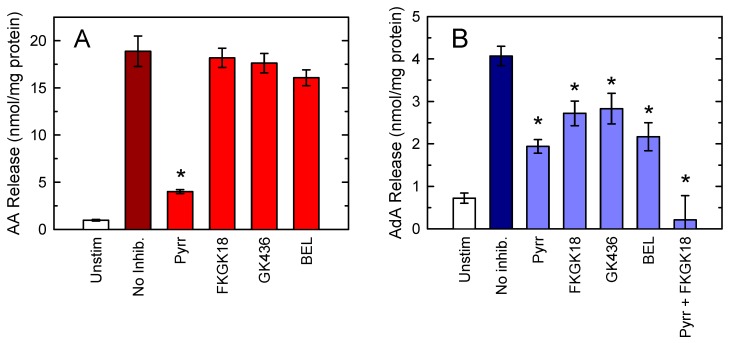
Release of AA and AdA by stimulated macrophages. The cells were either unstimulated (open bars) or stimulated by 1 mg/mL zymosan for 1 h (colored bars) in the absence (no inhibition) or presence of the following inhibitors: 2 µM pyrrophenone (Pyrr), 10 µM FKGK18, 5 µM GK436, 10 µM BEL, or 2 µM pyrrophenone plus 10 µM FKGK18 (see *x*-axis). Afterward, total content of AA (**A**) or AdA (**B**) was measured by GC/MS. The fatty acid release was calculated by subtracting the amount of phospholipid-bound AA or AdA in stimulated cells from that in unstimulated cells. Results are shown as mean values ± standard error of the mean. (n = 3). Note: * *p* < 0.05, significantly different from zymosan-stimulated cells in the absence of inhibitors.

In agreement with previous observations [22,24], pyrrophenone was found to almost completely inhibit zymosan-stimulated AA mobilization from phospholipids (Figure 4A). Importantly, pyrrophenone also partly inhibited AdA mobilization (Figure 4B). Conversely, none of the iPLA_2_-VIA inhibitors tested exerted appreciable effects on AA mobilization while significantly affecting AdA mobilization (Figure 4). The simultaneous addition of inhibitors of both cPLA_2_α and iPLA_2_-VIA resulted in complete inhibition of AdA mobilization (Figure 4B). These results suggest that unlike AA, AdA mobilization involves the action of both cPLA_2_α and iPLA_2_-VIA.

### 3.3. Studies with AdA-Enriched Cells

**Figure 5:**
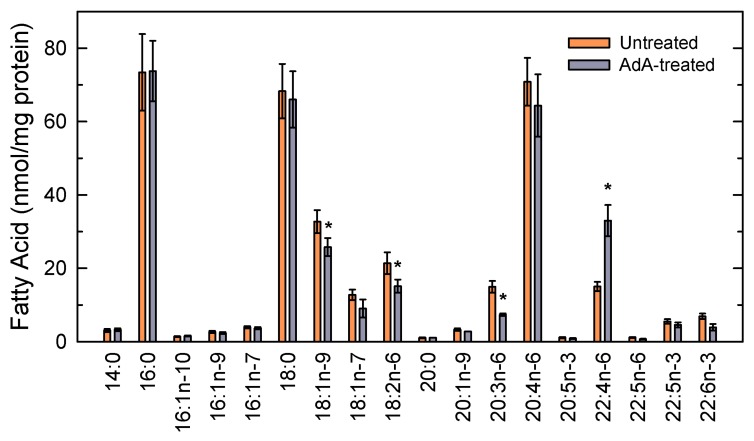
Fatty acid composition of murine peritoneal macrophages. The cells were either untreated (orange bars) or treated with exogenous AdA for 20 h (gray bars). Afterward, the total fatty acid profiles in cells were determined by GC/MS after converting the fatty acid glyceryl esters into fatty acid methyl esters. The fatty acids are designated by their number of carbon atoms, and after a colon, their number of double bonds. To differentiate isomers, the n − x (n minus x) nomenclature is used, where n is the number of carbons of a given fatty acid and x is an integer that gives the position of the last double bond of the molecule when subtracted from n. The data are expressed as mean values ± standard error of the mean of three individual replicates. Note: * *p* < 0.05, significantly different from incubations in the absence of AdA.

**Figure 6:**
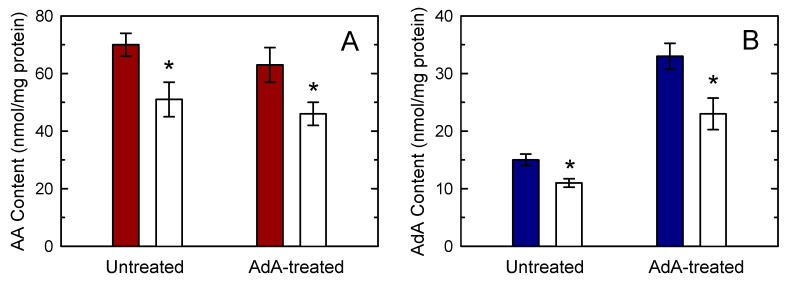
AA and AdA mobilization in zymosan-stimulated macrophages. The cells were either untreated or treated with exogenous AdA for 20 h (see x-axis). Afterward, the cells were unstimulated (colored bars) or stimulated (open bars) with 1 mg/mL zymosan for 1 h, and cellular AA content (**A**) or AdA content (**B**) was determined by GC/MS. Results are shown as mean values ± standard error of the mean (*n* = 3). Note: * *p* < 0.05, significantly different from unstimulated cells.

Given the structural similarities between AA and AdA, it could be envisioned that in some instances, AdA competes with AA for incorporation into phospholipids, which might result in reduced amounts of “mobilizable” AA within phospholipids, and hence reduced formation of pro-inflammatory eicosanoids [16,70,71]. To address this possibility, we incubated the macrophages with exogenous AdA (10 µM, 20 h), which resulted in the cells avidly incorporating the fatty acid into cellular phospholipids. These conditions led to a 2–3-fold increase in the amount of AdA esterified into phospholipids compared with untreated cells (33.2 ± 4.6 nmol per mg protein; mean ± standard error of the mean, *n* = 4). Analysis by GC/MS of the fatty acid content of the AdA-enriched cells revealed that the fatty acids typically displaced within phospholipids by AdA upon AdA supplementation were oleic acid (18:1n-9) and the n-6 series members linoleic (18:2n-6) and dihomo-γ-linolenic (20:3n-6) acids (Figure 5). Remarkably, little AA displacement occurred after AdA supplementation. Analysis of AA and AdA mobilization in zymosan-stimulated cells indicated that the extent of the AA response did not substantially differ from that observed from cells not treated with AdA, while AdA mobilization expectedly increased (Figure 6).

**Figure 7:**
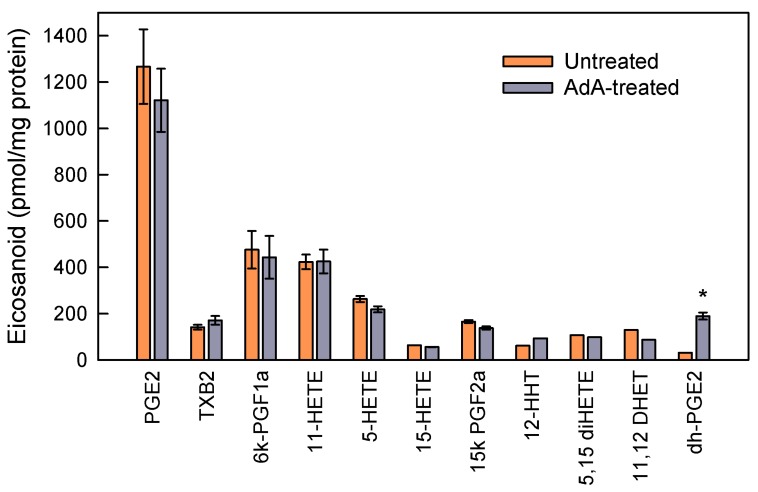
Eicosanoid production by stimulated macrophages. The cells were either untreated (orange bars) or treated with exogenous AdA for 20 h (gray bars). Afterward, the cells were stimulated by 1 mg/mL zymosan for 1 h, and eicosanoid content in the supernatants was analyzed by LC/MS. The data are expressed as mean values ± standard error of the mean of three individual replicates. Note: * *p* < 0.05, significantly different from incubations in the absence of AdA treatment. PGE2, prostaglandin E_2_; TXB2, thromboxane B_2_; 6k-PGF1a, 6-keto prostaglandin F_1__α_ (the stable product of prostaglandin I_2_); 11-HETE, 11-hydroxyeicosatetraenoic acid; 5-HETE, 5-hydroxyeicosatetraenoic acid; 15-HETE, 15-hydroxyeicosatetraenoic acid; 15k-PGF2a, 15-ketoprostaglandin F_2__α_; 12-HHT, 12-hydroxyheptadecatrienoic acid; 5,15-diHETE, 5,15-dihydroxyeicosatetraenoic acid; 11,12-DHET, 11,12-dihydroxyeicosatrienoic acid; dh-PGE2, dihomoprostaglandin E_2_.

Analysis of AA-derived metabolites produced by zymosan-stimulated cells did not appreciably change whether the cells had previously been treated with AdA or not, while the levels of the only AdA metabolite detected at significant levels in the stimulated macrophages, dihomoprostaglandin E_2_, increased in the AdA-treated cells (Figure 7). Collectively, these results suggest that in zymosan-activated macrophages, AdA does not influence AA metabolism leading to eicosanoid production.

## 4. Discussion

AdA is the 2-carbon elongation product of AA. Similar to AA, AdA can be mobilized from membrane phospholipids to serve as a substrate for the production of oxidized metabolites with a diverse array of biological functions [11,12,13,14,72]. We show in this work that AdA is present at significant quantities in the membrane phospholipids of murine peritoneal macrophages. The AdA-to-AA ratio in macrophages is 20–25%, which is similar to that found in other cells [13,14]. Stimulation of the macrophages with yeast-derived zymosan results in the cells releasing significant amounts of AdA, part of which is metabolized to dihomoprostaglandin E_2_. While macrophages appear to utilize AA for lipid mediator synthesis more efficiently than AdA, both qualitatively and quantitatively, our results support the concept that neither fatty acid competes with the other. Further, the data suggest that the processes leading to the mobilization and metabolism of both fatty acids are independently regulated. In support of these observations, enriching the cells with exogenous AdA has little effect on endogenous AA levels. AA release and metabolism in these AdA-enriched cells proceed in essentially the same way as in cells displaying normal levels of AdA. Consistent with these observations, studies by others have highlighted that under certain settings, some AdA-derived metabolites may display higher potency than their AA-derived counterparts, suggesting that they may serve non-redundant functions on their own [13,14,72,73,74,75]. Collectively, the results suggest that separate and selective mechanisms may exist for regulating AA and AdA utilization in macrophages.

Current evidence clearly indicates that cPLA_2_α is the major effector governing AA mobilization from membrane phospholipids in innate immune cells [55,76,77]. In contrast with the plethora of data regarding AA, no studies have been available to date identifying the PLA_2_ enzyme(s) responsible for effecting AdA release in activated cells. This work demonstrates the involvement of two PLA_2_ enzymes, namely cPLA_2_α and iPLA_2_-VIA, in agonist-induced AdA mobilization from activated macrophages, which contrasts with the sole involvement of cPLA_2_α in AA mobilization. This is a striking finding because a large number of studies have suggested that iPLA_2_-VIA does not participate, or plays a very limited role, in AA mobilization and attendant eicosanoid production in innate immunity and inflammation reactions [22,78,79,80,81]. The latter responses are almost completely suppressed in macrophages derived from cPLA_2_α-deficient mice compared with cells from wild-type mice [50].

In our studies, we have utilized yeast-derived zymosan particles. These are cell wall preparations from *S. cerevisiae* which have been extensively used as models for the induction of inflammatory responses to fungal infection [82]. Although zymosan activates many macrophage responses via engagement of toll-like receptor 2 (TLR2) [82], studies utilizing genetically-deficient mice have demonstrated that the receptor that couples fungal responses to cPLA_2_α activation and enhanced AA release are dectin-1 and dectin-2, not TLR2. [83,84]. Interestingly, macrophages exposed to the Gram-positive bacterium *L. monocytogenes* do release AA via engagement of TLR2 [85]. Stimulation of macrophages via TLR4 by lipopolysaccharide from Gram-negative bacteria promotes abundant AA mobilization in macrophage cell lines [86,87], but does so very poorly in primary macrophages [88]. Thus, multiple receptors on the surface of macrophages exist that can potentially induce cPLA_2_α activation and attendant AA release, and the involvement of one or another depends on the nature of the triggering stimulus. Future work should be aimed at defining whether, depending on stimulus, increased AdA mobilization also occurs through multiple macrophage receptors.

**Scheme 2:**
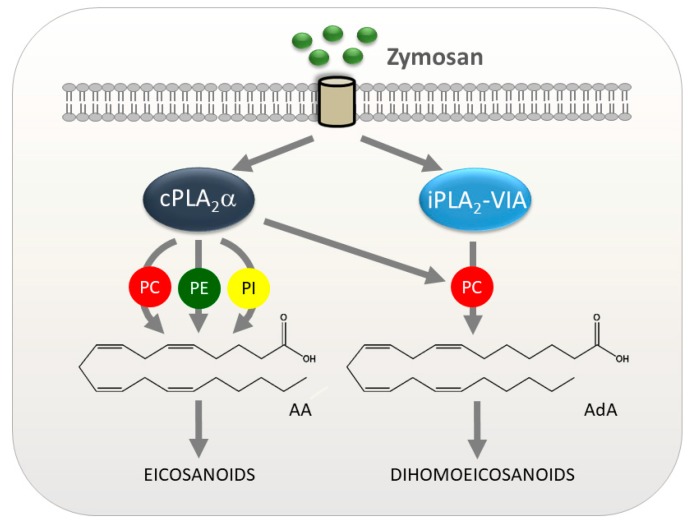
The scheme shows that cPLA_2_α and iPLA_2_-VIA are differentially involved in AA and AdA mobilization by zymosan-stimulated mouse peritoneal macrophages.

Although structurally related to AA, AdA does not have a double bond at C5, a potentially important feature for substrate recognition by cPLA_2_α [89]. In our study, cPLA_2_α inhibition by pyrrophenone blocks AA release to a larger extent than AdA release, which may reflect the higher affinity of cPLA_2_α for AA-containing phospholipids. It has been shown that cPLA_2_α has a deep and rigid channel-like active site that is able to accommodate a phospholipid substrate molecule in its entirety [90]. This confers the enzyme preference for phospholipids containing AA at the *sn*-2 position [90]. In contrast, iPLA_2_-VIA contains a more flexible and versatile active site; thus, this enzyme exhibits a more permissive specificity for the fatty acid at the *sn*-2 position [90]. In spite of these observations, our data using a live cell system show that cPLA_2_α is able to cleave both AA and AdA-containing substrates, while iPLA_2_-VIA cleaves only AdA-containing phospholipids. This raises the intriguing concept that in cells not only the inherent substrate specificity of each PLA_2_ determines phospholipid hydrolysis, and other factors should be taken into account. Among these, PLA_2_ accessibility to its substrate within the cell may profoundly limit free fatty acid release and attendant generation of biologically active mediators. Analysis of phospholipid molecular species containing either AA or AdA in the macrophages reflect no major differences (i.e., the major species containing AA also tend to be the major species containing AdA). However, there is a clear difference between the phospholipid pools used for the release of AA or AdA. While free AdA appears to proceed only from the hydrolysis of PC molecules, free AA appears to come from the hydrolysis of PC and PI, and perhaps PE as well. As discussed elsewhere [3,22,24,49], the lack of AA mobilization from PE probably reflects the involvement of CoA-independent transacylases, which rapidly restore the levels of AA in PE at the expense of AA-containing PC. In a similar vein, it could be argued that the lack of hydrolysis of AdA-containing PE could also be due to the replenishing action of CoA-independent transacylation reactions restoring AdA in PE. However, to the best of our knowledge, these reactions have not been shown to involve inositol phospholipids [91,92]. Thus, the lack of AdA hydrolysis from PI may likely reflect the inability of both cPLA_2_α and iPLA_2_-VIA to reach this particular substrate. Hence, while the AA-containing PI pool is accessible to phospholipase attack, the AdA-containing PI pool is not. Collectively, our results highlight fundamental differences between the mobilization of AA versus AdA in activated macrophages in terms of the PLA_2_ enzymes involved and the phospholipid class used as a substrate (Scheme 2). The data provide further support to the concept that intracellular substrate compartmentalization may limit the synthesis of fatty-acid-derived mediators.

## 5. Conclusions

Results from this study constitute an initial report addressing the mechanisms of AdA mobilization from membrane phospholipids in cells of innate immunity and inflammation. We demonstrated that AdA mobilization is mediated by two different PLA_2_s, namely cPLA_2_α and iPLA_2_-VIA (Scheme 2). Further studies will be needed to precisely determine the contribution of each PLA_2_ to overall AdA release under different stimulation conditions, and to assess whether cross-talk exists between the two enzymes. Elucidation of the innate immune functions of AdA and its metabolites may not prove to be an easy task, because inhibitors that block cPLA_2_α or AdA metabolism via cyclooxygenase, lipoxygenase, or CYP450 pathways will also affect AA and its metabolism. Given the sole involvement of iPLA_2_-VIA in AdA release but not in AA release, selective inhibition of this PLA_2_ could be considered to evaluate the effect of altering AdA metabolism on the outcome of immunoinflammatory diseases.

## Abbreviations

AA: arachidonic acid; AdA: adrenic acid; cPLA_2_α: group IVA Ca^2+^-dependent cytosolic phospholipase A_2_α; GC/MS: gas chromatography coupled to mass spectrometry; iPLA_2_-VIA: group IVA Ca^2+^-independent phospholipase A_2_; LC/MS: liquid chromatography coupled to mass spectrometry; PC: choline-containing phospholipids; PE: ethanolamine-containing phospholipids; PI: phosphatidylinositol; PLA_2_: phospholipase A_2_; PS: phosphatidylserine.
